# Description of the parasitic fauna of a specimen of *Didelphis albiventris* at Rio Grande do Sul

**DOI:** 10.29374/2527-2179.bjvm000524

**Published:** 2024-04-02

**Authors:** Julia Somavilla Lignon, Diego Moscarelli Pinto, Silvia Gonzalez Monteiro, Natália Soares Martins, Julia Victória de Souza, Giulia Ribeiro Meireles, Tamires Silva dos Santos, Felipe Geraldo Pappen, Fábio Raphael Pascoti Bruhn

**Affiliations:** 1 Veterinarian, Laboratório de Epidemiologia Veterinária, Departamento de Veterinária Preventiva, Universidade Federal de Pelotas (UFPel), Pelotas, RS, Brazil; 2 Veterinarian, Laboratório do Grupo de Estudos em Enfermidades Parasitárias, Departamento de Veterinária Preventiva, UFPel, Pelotas, RS, Brazil; 3 Veterinarian, Laboratório de Parasitologia Veterinária, Departamento de Microbiologia e Parasitologia, Universidade Federal de Santa Maria, Santa Maria, RS, Brazil

**Keywords:** Coproparasitological, endoparasites, wildlife, marsupial, zoonosis, Coproparasitológico, endoparasitos, animais silvestres, marsupial, zoonoses

## Abstract

*Didelphis albiventris* is considered the most common marsupial in Rio Grande do Sul. With omnivorous and synanthropic habits, it can serve as a host to various parasites, playing an important role in maintaining their biological cycle. Despite being a widespread and abundant species, it has a relatively little-known parasitic fauna. Therefore, the aim of this study was to report the diversity of parasites in a fecal sample from *D. albiventris* in Rio Grande do Sul, Southern Brazil. Modified Centrifugal-flotation and Spontaneous sedimentation techniques were used, revealing a high taxonomic diversity of parasites. Eggs of *Ancylostoma* spp., *Toxocara* spp., and Anoplocephalidae were reported for the first time in the host in the southern region of the country, along with the first report of pseudoparasitism by *Syphacia* spp. and *Monocystis* spp. in this animal species. The presence of different parasites in the feces of *D. albiventris* is of utmost importance, primarily for public health, but also for understanding the biodiversity of parasites present in wildlife, which has been poorly studied until now. This allows the implementation of effective strategies for controlling, preventing and treating these diseases.

## Resumo

*Didelphis albiventris* é considerado o marsupial mais comum no Rio Grande do Sul. Com hábitos onívoros e sinantrópicos, pode servir de hospedeiro para diversos parasitas, desempenhando importante papel na manutenção do seu ciclo biológico. Apesar de ser uma espécie difundida e abundante, possui uma fauna parasitária relativamente pouco conhecida. Portanto, o objetivo deste estudo foi relatar a diversidade de parasitas em uma amostra fecal de *D. albiventris* no Rio Grande do Sul, Sul do Brasil. Foram utilizadas técnicas modificadas de flutuação centrífuga e sedimentação espontânea, revelando uma alta diversidade taxonômica de parasitas. Ovos de *Ancylostoma* spp., *Toxocara* spp. e Anoplocephalidae foram relatados pela primeira vez no hospedeiro na região sul do país, juntamente com o primeiro relato de pseudoparasitismo por *Syphacia* spp. e *Monocystis* spp. nesta espécie animal. A presença de diferentes parasitas nas fezes de *D. albiventris* é de extrema importância, principalmente para a saúde pública, mas também para a compreensão da biodiversidade de parasitas presentes na vida selvagem, que tem sido pouco estudada até agora. Isto permite a implementação de estratégias eficazes para controlar, prevenir e tratar estas doenças.

## Introduction

*Didelphis albiventris* (Lund, 1840), commonly known as the “white-eared opossum” or “saruê,” is a marsupial species frequently encountered in countries such as Argentina, Paraguay, Uruguay, Bolivia, Brazil, Guyana, Suriname, and the south of Venezuela (Tocchio et al., 2014). In Brazil, *D. albiventris* has been documented in states such as Bahia, Mato Grosso, Mato Grosso do Sul, Maranhão, Paraná, Rio Grande do Sul, Santa Catarina, and São Paulo (Antunes & Brum, 2005).

Exhibiting nocturnal and omnivorous behaviors, opossums primarily feed on fruits, seeds, shoots, as well as invertebrates and small vertebrates, including little birds, amphibians, reptiles, and mammals, in their natural habitat. Consequently, they are considered opportunistic feeders based on food availability. The extensive dietary diversity of *D. albiventris* may be linked to its high degree of synanthropism, showcasing its effective adaptation to environments shaped or altered by human activities (Antunes & Brum, 2005).

Owing to these characteristics, opossums play a significant role in the epidemiology of parasitic diseases, acting as potential vectors of pathogenic agents among wild and domestic animals, including humans. This situation is exacerbated by their increasingly frequent presence in peri-urban and urban areas (Bezerra-Santos et al., 2020a; Lignon et al., 2023).

Studies focusing on parasites in wild animals are crucial for investigating the biodiversity of species affecting them and assessing the potential risks posed by these parasites to public health (Martins et al., 2004). However, for many wild species, comprehensive studies are lacking. Even for widespread and abundant species like *D. albiventris*, the parasitic fauna remains relatively understudied.

Therefore, the objective of this study was to document the diversity of parasites identified in the feces of a white-eared opossum (*D. albiventris*) at Rio Grande do Sul (RS), Southern Brazil.

## Case report

A faecal sample of adult male white-eared opossum (*D. albiventris*), was received for parasitological diagnosis, in the laboratory of the Grupo de Estudos em Enfermidades Parasitárias, Faculdade de Veterinária, from the Universidade Federal de Pelotas (UFPel), located in Capão do Leão, RS. The animal was found on the side of the highways in the city of Pelotas, RS, victim of being run over, and was being treated at the Hospital Clínico Veterinário (HCV) at UFPel.

The sample underwent processing following modified Zinc Sulfate Flotation Centrifuge techniques, as outlined by Monteiro (2017), and Spontaneous sedimentation (Hoffman et al., 1934). For identification purposes, all structures enabling the identification or differentiation of eggs at their smallest possible taxon, such as characteristics and shell ornaments, embryonic and larval formations, and the presence of operculum and aculea, were utilized. Identification was carried out by comparing the morphometry observed with that of species previously described in the literature for the host species, as described by Abdel-Gaber (2016), Aragón-Pech et al. (2018), Prado et al. (2019), Teodoro et al. (2019) and Bezerra-Santos et al. (2020b), using an Olympus optical microscope (CX22 series).

**Figure 1:**
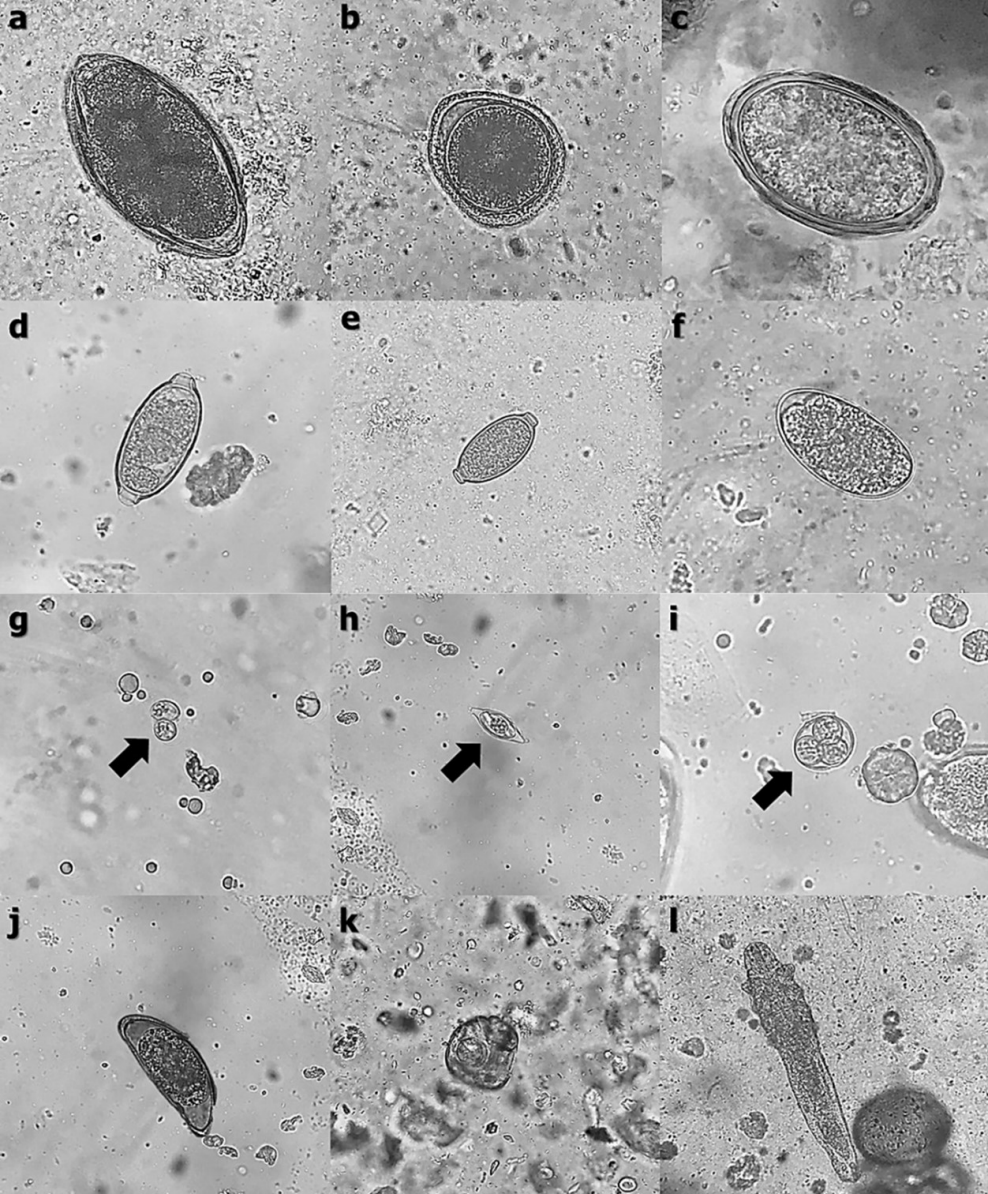
Eggs, oocysts, sporocysts and mite found in fecal sample of *Didelphis albiventris* in southern Brazil. (a) *Cruzia* spp. egg; (b) *Toxocara* spp. egg; (c) *Aspidodera* spp. egg; (d) *Trichuris* spp. egg; (e) *Capillaria* spp. egg; (f) *Ancylostoma* egg; (g) *Sarcocystis* spp. sporocysts (arrow); (h) *Monocystis* spp. sporocysts (arrow); (i) *Eimeria* spp. oocysts (arrow); (j) *Syphacia* egg; (k) Anoplocephalid egg; (l) *Demodex* spp. 400X magnification.

Through the examination of *D. albiventris* feces, a considerable taxonomic diversity of parasites was observed, including new records, illustrated in Figure 1. In total, eggs of seven nematodes and one cestode, three protozoa, and one mite were identified, corresponding to: *Cruzia* spp. egg (Figure 1a); *Toxocara* spp. egg (Figure 1b); *Aspidodera* spp. egg (Figure 1c); *Trichuris* spp. egg (Figure 1d); *Capillaria* spp. egg (Figure 1e); *Ancylostoma* egg (Figure 1f); *Sarcocystis* spp. oocysts (Figure 1g); *Monocystis* spp. sporocysts (Figure 1h); *Eimeria* spp. oocysts (Figure 1i); *Syphacia* egg (Figure 1j); Anoplocephalid egg (Figure 1k) and *Demodex* spp. (Figure 1l).

## Discussion

Parasites such as *Cruzia* spp., *Aspidodera* spp., *Trichuris* spp., and *Capillaria* spp. have been previously documented in this host within the same region, as indicated by Antunes and Brum (2005). However, nearly 20 years have elapsed, and despite our understanding of the diverse gastrointestinal parasitic fauna in *Didelphis* species — comprising a wide array of helminths (Chero et al., 2017; Aragón-Pech et al., 2018) and identified protozoa (Zanette et al., 2008) — there remains limited knowledge about them in southern Brazil, particularly in RS.

*Ancylostoma* spp. and *Toxocara* spp. were previously identified in *D. albiventris* in the state of Alagoas, northeastern Brazil (Silva et al., 2017). Parasites of the family Anoplocephalidae (e.g., *Anoplocephala* sp.) were recorded in *Didelphis virginiana* by Krupp and Quillin (1964). Now, both are reported for the first time affecting the study host (*D. albiventris*) in RS. The presence of eggs from these parasites in the sample may be linked to the increasingly frequent presence of opossums in peri-domestic and domestic environments, where niches between mammal species (wild and domestic) overlap, facilitating the infection of these marsupials. Some of these parasites, including *Ancylostoma* spp., *Toxocara* spp., *Trichuris* spp., and *Capillaria* spp., as well as some species of Anoplocephalidae (e.g., *Bertiella* spp.), are potentially zoonotic (Denegri et al., 1998; Monteiro, 2017). Therefore, the risk of their spread and infection in humans should not be neglected.

The discovery of *Sarcocystis* spp. oocysts, previously described by Lins et al. (2011) in the studied location, underscores the caution needed for equine farms in the region. Due to the omnivorous habits of opossums, they tend to approach animal breeding sites in search of food, potentially contaminating them with oocysts and protozoan sporocysts. Opossums act as definitive hosts for *Sarcocystis neurona*, although clinical disease is rarely observed in them. Nevertheless, the agent can cause the Equine Protozoal Myeloencephalitis (EPM) — a significant neurological disease with primary clinical signs of motor incoordination, decreased proprioception, muscle weakness, muscular atrophy, and cranial nerve palsy (Silva, 2003). According to Reed et al. (2016), the presence of possums in equine breeding environments increases the risk of EPM by up to 2.5 times. This underscores the importance of maintaining hygiene practices in breeding facilities to control the disease, ensuring cleanliness and proper food storage.

Among the known parasites in *Didelphis*, *Eimeria* spp. are commonly found (Duszynski, 2016). While most species in the genus were once considered to exhibit strict specificity toward their hosts, phylogenetic, cross-transmission, and morphological studies have demonstrated the ability of some to infect different host species within the same genus or even species in different host families (Vrba & Pakandl, 2015; Duszynski, 2016). Fehlberg et al. (2018) and Bezerra-Santos et al. (2020b) recently reported different species of *Eimeria* spp. in *Didelphis aurita*, in the northeast and southeast regions of Brazil. In contrast, Zanette et al. (2008) reported *Eimeria* oocysts in *D. albiventris* in the Central region of RS. Here, we report for the first time the occurrence of *Eimeria* spp. in *D. albiventris* in the southern part of the state.

The discovery of mites such as *Demodex* spp. in the feces of *D. albiventris* can be explained because these ectoparasites, at all stages, can be found in lymph nodes, the intestinal wall, spleen, liver, kidneys, bladder, lung, thyroid, blood, urine, and feces, as well as in the dermis (inside the hair follicle and sebaceous glands). However, when observed in these extracutaneous organs, they are generally dead and degenerated, representing drainage to any of these areas via the blood or lymphatic stream of the infested animal (Muller et al., 1985; Monteiro, 2017). To date, there have been no reports in the literature involving *Demodex* parasitism in these hosts.

Parasites belonging to the Oxyuridae family, such as *Syphacia* spp., are the most common intestinal helminths in rodents (Robles & Navone, 2007; Abdel-Gaber, 2016). However, due to the omnivorous habits of opossums the authors believe that this is the first description of pseudoparasitism by eggs of this nematode in opossums, as reported by Moraes et al. (2019) in coatis (*Nasua nasua*). In addition to the negative influence on the health of rodents, the zoonotic potential of some members of the Oxyuridae family was also confirmed. Reports of human infection by *Syphacia* spp. were described by Riley (1919), Stone and Manwell (1966), Mahmoud et al. (2009), and later also by Taylor et al. (2017). Therefore, opossums can contribute to the spread of this parasite in the environment, increasing the risk of infection in mammals, including humans.

Furthermore, we describe the first case of pseudoparasitism by *Monocystis* spp. sporocysts in opossums. These agents are protozoa from the phylum Apicomplexa, exclusively affecting the seminal vesicles and promoting infertility in earthworms. Earthworms become infected by ingesting oocysts in the soil, and when ingested by vertebrates, they release sporocysts, which are excreted along with the feces of the latter, making them accidental hosts (Field and Michiels, 2006). Although pseudoparasitism by this protozoan has been reported in other animals such as coatis (Moraes et al., 2019) and nine-banded armadillos (*Dasypus novemcinctus*) (Prado et al., 2019), there is no knowledge about its pathogenic effect on vertebrate hosts. This occurrence is usually associated with the eating habits of these individuals (Blagburn, 2010). As mentioned earlier, opossums have omnivorous habits, and their diet includes small vertebrates, invertebrates (e.g., annelids), as well as fruits and seeds (Antunes & Brum, 2005). Additionally, understanding the morphology and life cycle of pseudoparasites allows for avoiding false-positive diagnoses, especially because in coproparasitological examinations that involve fecal flotation, the sporocyst is similar in appearance to *Trichuris* spp. eggs, although very small. Furthermore, when considering animal populations, especially those with a high density of individuals, identifying these parasites in the feces of individuals can lead to a misunderstanding of the necessity to treat these animals (Prado et al., 2019). This situation is exacerbated by the scarcity of literature on parasitic agents in wild animals, emphasizing the need to scientifically describe new findings in these animals.

Understanding the parasitic fauna of wild species is crucial for several reasons. It not only contributes to the general knowledge of biodiversity, but also plays a significant role in maintaining the health of ecosystems. Knowledge about parasites in wild animals is vital for public health, especially when considering potential zoonotic parasites that can affect humans. The findings of this report can help in the development of effective strategies for control and prevention, as well as treatment, of diseases of wildlife and the human population.

## Conclusion

In this study, we report for the first time the finding of eggs of *Ancylostoma* spp., *Toxocara* spp., and Anoplocephalidae in the host at Rio Grande do Sul, Southern region of Brazil, as well as the first report of pseudoparasitism by *Syphacia* spp. and *Monocystis* spp. in this animal species. The presence of different parasites in the feces of *D. albiventris* is of utmost importance, primarily for public health but also for understanding the biodiversity of parasites present in wildlife, which has been poorly studied until now. This allows the implementation of effective strategies for controlling, preventing and treating these diseases.
